# Association between dietary diversity with overweight and obesity: A cross-sectional study conducted among pastoralists in Monduli District in Tanzania

**DOI:** 10.1371/journal.pone.0244813

**Published:** 2021-01-13

**Authors:** Ahmed Gharib Khamis, Julius Edward Ntwenya, Mbazi Senkoro, Sayoki Godfrey Mfinanga, Katharina Kreppel, Akwilina Wendelin Mwanri, Bassirou Bonfoh, Gideon Kwesigabo

**Affiliations:** 1 Department of Epidemiology and Biostatistics, Muhimbili University of Health and Allied Sciences, Dar-es-Salaam, Tanzania; 2 Department of Public Health, The University of Dodoma, Dodoma, Tanzania; 3 National Institute for Medical Research, Muhimbili Centre, Dar-es-Salaam, Tanzania; 4 School of Life Sciences and Bio-Engineering, Nelson Mandela African Institution of Science and Technology, Arusha, Tanzania; 5 Department of Environmental Health and Ecological Sciences, Ifakara Health Institute, Dar-es-salaam, Tanzania; 6 Department of Food Technology, Nutrition and Consumer Sciences, Sokoine University of Agriculture, Morogoro, Tanzania; 7 Centre Suisse de Recherches Scientifiques en Côte d'Ivoire, Abidjan, Côte d'Ivoire; University of Arkansas for Medical Sciences, UNITED STATES

## Abstract

**Background:**

The prevalence of overweight and obesity is rising at a rapid pace and is associated with negative health consequences like cardiovascular diseases, type 2 diabetes and cancer. Obesity is a multifactorial problem that develops mainly from lifestyle factors including physical inactivity and poor dietary intake. Dietary diversity is a simplified method for assessing the adequacy and quality of diet and is associated with nutritional need and overall health status. Therefore, we conducted this study to synthesize the associations between consumption of a diversified diet and overweight/ obesity among adults living in pastoral communities in Monduli district in Tanzania.

**Methods:**

This was a cross-sectional study conducted among 510 adults aged ≥ 18 years old in the Monduli district, Arusha region in Tanzania. We conducted face-to-face interviews to collect information about socio-demographic characteristics, 24-hours dietary recall, and anthropometric measurements. The dietary diversity score (DDS) was constructed and used to determine the diversity of the diet consumed. We performed the multivariate Poisson regressions to determine the prevalence ratio (PR) with 95% confidence intervals (CI). The dependent variables were overweight and general obesity as measured by body mass index (BMI), abdominal obesity measured by waist-hip ratio (WHR) and waist circumference (WC).

**Results:**

The prevalence of general obesity based on BMI was 20.2% (95%CI; 16.9–23.9), abdominal obesity based on WHR was 37.8% (95%CI; 33.7–42.1), and WC was 29.1% (95%CI; 25.2–33.1). More than half (54.3%) of the participants consumed an adequate dietary diversity (DDS ≥4). After adjustment for potential confounders, the prevalence of abdominal obesity by WHR decreased with higher DDS among male (APR = 0.42; 95% CI, 0.22–0.77) and female participants (APR = 0.63; 95% CI, 0.41–0.94). There were inconsistent positive associations between DDS and prevalence of overweight and general obesity among male and female. There was no association between DDS and abdominal obesity by WC.

**Conclusion:**

More than half of the pastoralists have consumed an adequate diversified diet. Given the inconsistent findings on associations between dietary diversity and obesity measures, this study suggests that targeting dietary diversity as an overweight/obesity prevention strategy requires careful consideration.

## Introduction

Obesity is a growing public health problem worldwide and is associated with several types of non-communicable diseases (NCDs) such as type 2 diabetes, cancer and cardiovascular diseases [1, 2]. Globally, the rising burden of overweight and obesity and the associated comorbidities is calling for evidence-based actions [3]. The World Health Organization (WHO) estimated that 1.9 billion adults worldwide were overweight and more than 600 million were obese in 2014 [4]. Most of the low and middle income countries (LMICs) are generally considered to be affected by rapidly increasing prevalence of obesity and overweight while decreasing in underweight [2]. Tanzania, while still fighting malnutrition, is also facing increasing rates of obesity [5]. The recent national nutrition survey reported that the prevalence of overweight or obesity among adult females has nearly tripled from 11.3% in 1991 to 31.7% in 2018 [5]. The increase in obesity is highly associated with modern lifestyle habits including dietary intake, physical inactivity, and their interactions with genetic and environmental factors [6].

There are various studies showing that pastorals areas are at a higher risk of malnutrition and various diseases due to challenges in their daily food production, livelihoods or mobility. Such vulnerabilities may likely result in high food insecurity and poor health outcomes. A previous study conducted among pastoralists in Simanjiro district in Tanzania suggested that their diet consists mainly of starchy staples with little fruits and vegetables [7]. Moreover, others have reported that their meal pattern is mainly composed of animal-sourced foods like meat and milk which might be linked to obesity and other associated NCDs [8–12]. Therefore, given the low dietary diversity and other lifestyle risk factors of rural and urban populations are highly prevalent in Tanzania [13]. It is critical to understand the association between dietary diversity practice of the pastoralists with overweight and obesity in order to set up evidence-based actions for the prevention of diet-related NCDs.

Dietary diversity score (DDS) is a simplified method for assessing the quality of overall diets [14]. Assessing the quality of diet using DDS is simple and inexpensive, with little methodological challenges. It is defined as a simple count of food items or food groups consumed over a certain time. It is also considered as a potential proxy indicator to reflect adequacy in nutrient intake and calorie consumption [15–18]. The role of dietary diversity in preventing obesity and other metabolic diseases has drawn greater attention in recent literature because it reflects the multiple components of the diet rather than single nutrients [15]. Additionally, studies are showing that dietary diversity may be an important component to be considered in terms of NCDs prevention [19].

To date, numerous studies have been conducted to examine the role of dietary diversification on obesity and other health markers among various populations in different countries [20–29]. For-example, dietary diversity was found to be associated with general obesity and abdominal obesity in Asian and Arabic countries [20, 21, 27]. Some studies have shown that dietary diversity is inversely associated with metabolic syndromes [22], while others failed to see an association [28]. A systematic review and a meta-analysis on the associations between dietary diversity and obesity measures were inconclusive [24, 29], possibly due to inconsistency in the methods used to determine dietary diversity and/or the population of interest. Diet may affect sub-populations differently, warranting the need to understand the diet-disease relationship of specific target population. Based on our literature review, numerous studies have been conducted among urban populations, while little to no attention has been given to rural and pastoral sub-populations, particularly in Tanzania.

Considering the above, the overall objective of this study was to examine the association between consumption of diversified diet on overweight and obesity measures among adults living in pastoral communities in Monduli district in Tanzania. Exploring the above associations could help government and implementing partners to develop food based dietary guidelines, inform policy formulation related to obesity prevention and management, and to fill the gap of evidence based NCDs interventions in the country.

## Materials and methods

### Study design and setting

This is a cross-sectional study conducted among adults aged 18 years old and above in the Monduli district from November 2019 to February 2020. This district is one of the five other districts under the region of Arusha in northern part of Tanzania. This district was purposely selected because it is known to be the homeland for pastoralist communities whose livelihood depends on livestock keeping [11]. The district covers an area of about 6,993 square kilometers, and has an estimated population of 123,153. The study participants were permanent residents from five villages in the district namely; Emaeret, Enguik, Mto wa mbu, Monduli juu and Esilalei; who were recruited in their traditional homestead (bomas). The major ethnic group inhabiting in those villages are the “Maasai” people (97%) who are traditionally practicing pastoralism, and majority of them practiced semi-nomadism. The remaining few ethnic groups live mostly in the town areas of the district. Apart from livestock keeping, people engage in cultivation of major crops including maize, beans, paddy, wheat/barley, banana and coffee [10]. We calculated the sample size by assuming obesity prevalence of 22% in a similar setting [7], 5% precision at 95% confidence level, 5% non-response rate, and design effect of 1.5, which results in a sample size of 474. We conducted complete interviews with 510 participants. Multistage cluster sampling method utilizing the administrative structure as the sampling frame was used to select participants. We obtained a list of all villages from the Monduli district office. Five (5) out of 62 listed were randomly selected and their respective village chairperson provided a household sampling frame. The number of households from each village was selected randomly proportionate to the village sizes. All adults in the household meeting the inclusion criteria were invited to participate in the study. Participants were eligible if they were 18 years or older, were permanent residents of the selected villages for at least 6 months, able to provide informed consent, and willing to follow the study procedures, as described previously [30]. The data were collected together with research assistants (RAs) who were trained health professionals. All RAs received trainings for three days by a principal investigator before data collection. All of them spoke Swahili and the traditional Maasai language that were used during interviews. The data were directly captured in mobile phones and tablets using Open Data Kit software (ODK collect version 1.10) modified for closed and open-ended questions (**S1 Questionnaires**). The study procedures were approved by the Ethics Committee of the Muhimbili University of Health and Allied Sciences (MUHAS) approval number MUHAS-REC-9-2019-038. Permission to start the study was given by the Monduli District Medical Officer (DMO). Informed consents were obtained from all participants and from the village head.

### Anthropometric measurements

Weight and height measurements were taken using a digital weighing machine (SECA 813, USA) and a stadiometer, respectively. Body weight was measured to the nearest 0.1 kg precision. Waist and hip circumferences were measured using a non-stretchable measuring tape. Measurements were repeated at least two times for accuracy. We calculated body mass index (BMI) as weight (kilograms) divided by squared height (meters) and then categorized as per World Health Organization (WHO) standards of < 18.5 kg/m^2^ as underweight, 18.5–25 kg/m^2^ as normal, 25–30 kg/m^2^ as overweight, and above 30 kg/m^2^ as obesity. Waist circumference (WC) was measured at the thinnest point of the abdomen at the end of a normal expiration. The hip circumference was measured at the maximum circumference over the buttock horizontally. Both waist and hip circumferences were measured using a measuring tape with 1 mm accuracy. Waist-hip ratio (WHR) was calculated by dividing the waist by the hip circumference. Abdominal obesity by WHR was defined based on the WHO cut-offs, whereby measurement of WHR≥ 0.90 in males and WHR≥ 0.85 in females were regarded as having obesity [31]. Furthermore, abdominal obesity by WC was determined by using a cut-off point of ≥ 102 cm for males and ≥ 88 cm for females, as per the WHO recommendation [31].

### Dietary diversity determination

To collect the dietary intake information, we asked all participants to recall their previous 24-hours foods and beverages consumed including foods eaten outside the home. A dietary diversity score (DDS) was constructed according to the Food and Agriculture Organization (FAO) guidelines for individual dietary diversity [14]. To calculate the DDS, reported food items were categorized into 12 food groups which are (i) cereals; (ii) legumes; (iii) roots and tubers; (iv) vegetables (v); fruits (vi) milk; (vi) eggs; (vii) meat; (viii) fats; (ix) fish; (xi) sweets and (xii) beverages and condiments. Consumption of each food group was transformed into binary variables (1 = Yes, 0 = No) to indicate whether food items from a particular group was eaten or not. Then, aggregated DDS was created by summing the number of food groups reported by each participant (possible range from 0 to 12). A DDS of less than four (4) food groups was regarded as low or inadequate dietary diversity. Similar score was used to classify inadequate dietary diversity in other study [32]. Finally, DDS was divided into terciles (Lower, middle and higher) for analysis.

### Independent variables

Information on age, education, physical activity, morbidity and income were self-reported by each participant. Age was categorized into four groups: 18–29 years (young), 30–39 years (middle-aged adults), 40–59 (matured adult) and 60 years or above (old adults). Educational attainment was divided into five common groups following the school system in Tanzania. The groups were; no education, primary education, secondary education (O-level), advanced secondary education (A-level) and college or University. Monthly income was defined as total earnings for the subjects in the previous month and was expressed in Tanzania shillings (Tsh); less than 50,000 (about 22 USD), between 50,000 to 200,000, and above 200,000. Smokers were defined as subjects who had responded “Yes” to the question “Have you ever smoked/used tobacco product in your entire life? Alcohol consumption was categorized into: Never, occasional (one to three drinks per week), and frequent (more than three drinks per week). Morbidity information was collected from all participants by asking them to report if they had diarrhea, were diagnosed with malaria, and/or helminth infections in the past 30 days before the interview.

Moreover, physical activity was assessed by using the Global Physical Activity Questionnaire (GPAQ version 2) adopted from the WHO [33]. This was done by asking participants to recall and estimate the amount of time they spend doing different types of activity including working, walking, sports and recreational. The physical activity level was measured by metabolic equivalents (MET) in minutes per week and divided into three groups: inactive (≤599 MET-min/week), moderate active (600–2999 MET-min/week), highly active (≥3000 MET-min/week) based on GPAC classification [7].

### Statistical analysis

All variables were cleaned in Stata (Stata 13/SE, StataCorp, College Station, TX) and analyzed using the Statistical Package for Social Sciences (Version 23.0; IBM Corp., Armonk, NY, USA). Frequency, percentages were used to describe the characteristics of the study population. The characteristics were compared between obesity prevalence using Chi-square test. The outcome variables of interest were overweight and obesity measures using body mass index (BMI), abdominal obesity based on waist-hip ratio (WHR) and abdominal obesity based on waist circumference (WC). All outcome variables were categorized into binary (1 = Obesity, 0 = No obesity) for regression analysis. Prevalence ratio (PR) was estimated from Poisson regression with robust variance to have a better estimate of the associations. This is because all outcomes are common with high prevalence of more than 10%. First, DDS tercile (Lower, middle, and higher) was used as an independent variable to examine the crude association with overweight and obesity measures. Then, variables were entered in the multivariate models with DDS tercile variable to identify the adjusted associations by controlling confounders. As reported in the previous literature [20, 32], various characteristics were used control for possible confounders in the models. Model 1 was adjusted for biological factor of age. Model 2 was adjusted for socio-economic and physical activity factors. Model 3 was adjusted for all possible confounding factors. Associations were examined in gender-specific regression models. All crude prevalence ratios (PR) and adjusted prevalence ratios (APR) of overweight and obesity with 95% confidence intervals (CIs) were two-sided and considered significant if p≤0.05.

## Results

### Characteristics of the study population

A total of 510 subjects were enrolled in this study with the majority are females (52.7%) than males (47.3%). Their median (IQR) age is 36 (52–25) with majority (37.3%) were aged between 18–29 years. Most of them (52.1%) completed primary education; few (6.5%) reached the university/college level. About 28.4% of the participants reported an average monthly income of less than 50,000 Tsh, and 27.4% have no income. Different types of illness were self-reported especially diarrhea (5.5%), followed by malaria (4.3%), and helminth infections (2.2%). The prevalence of frequent alcohol drinkers (25.3%) was higher than that of smokers (12.2%). More than 40% were physically inactive according to criteria applied. The general obesity prevalence was 20.2% (95%CI; 16.9–23.9), overweight was 24.7% (95% CI; 21–28), abdominal obesity by WHR was 37.8% (95%CI; 33.7–42.1), and abdominal obesity by WC was 29.1% (95%CI; 25.2–33.1). The prevalence of obesity increases significantly with increasing in age of the study population (*p*<0.001). In general, females have significantly higher prevalence of obesity than males (*p*<0.001). In addition, the prevalence of general obesity (58.3%) and abdominal obesity by WC (54.1%) were significantly higher among inactive than highly active participants. Similarly, those with less or no income have higher prevalence of obesity than those with higher income across all measures, as shown in **Table 1.**

**Table 1:** Characteristics of the participants according to obesity measures in Monduli district, Tanzania (*N* = 510).

	Overall	Obesity(BMI)^*b*^	*P*-value^a^	Obesity(WHR)^c^	*P*-value^a^	Obesity(WC)^d^	*P*-value^a^
Variables	N = 510	N = 103		N = 193		N = 148	
	n(%)	n(%)		n(%)		n(%)	
**Age (years)**							
*Median (IQR)*	*36(52–25)*						
18–29	190(37.3)	20(19.4)	**<0.001**	36(18.7)	**<0.001**	23(15.5)	**<0.001**
30–39	92(18.0)	20(19.4)		26(13.5)		28(19)	
40–59	139(27.3)	40(38.8)		68(35.2)		48(32.4)	
Above 60	89(17.4)	23(22.3)		63(32.6)		49(33.1)	
**Gender**							
Male	241(47.3)	6(5.8)	**<0.001**	64(33.2)	**<0.001**	9(6.1)	**<0.001**
Female	269(52.7)	97(94.2)		129(66.8)		139(93.9)	
**Alcohol consumption**							
Never	358(69.4)	67(65.1)	**0.516**	134(69.4)	**0.943**	105(70.9)	**0.711**
Occasional	27(5.3)	7(6.8)		11(5.7)		6(4.1)	
Frequent	129(25.3)	29(28.1)		48(24.9)		37(25)	
**Smoking**							
Ever smoke	62(12.2)	3(2.9)	**0.001**	22(11.4)	**0.683**	2(1.35)	**<0.001**
Never smoke	448(87.8)	100(97.1)		171(88.6)		146(98.6)	
**Malaria**							
Yes	22(4.3)	2(1.9)	**0.186**	6(3.1)	**0.296**	5(3.4)	**0.506**
No	488(95.7)	101(98.1)		187(96.9)		143(96.6)	
**Helminthes infections (worms)**							
Yes	11(2.2)	2(1.9)	**0.866**	4(2.1)	**0.919**	4(2.7)	**0.587**
No	499(97.8)	101(98.1)		189(97.9)		144(97.3)	
**Diarrhoea**							
Yes	28(5.5)	10(9.7)	**0.035**	16(8.3)	**0.030**	13(8.8)	**0.037**
No	482(94.5)	93(90.3)		177(91.7)		135(91.2)	
**Income (Tsh)**							
*Median (IQR)*	*70*,*000(200*,*000–30*,*000*						
No income	140(27.4)	31(30.1)	**0.033**	61(31.6)	**0.010**	46(31.1)	**0.003**
<50,000	145(28.4)	36(34.9)		65(33.7)		46(31.1)	
50,000–200,000	100(19.6)	10(9.7)		28(14.5)		14(9.5)	
>200,000	125(24.5)	26(25.2)		39(20.2)		42(28.4)	
**Educational attainment**							
No education	110(21.6)	18(17.5)	**0.116**	58(30.1)	**<0.001**	38(25.7)	**0.361**
Primary education	266(52.1)	50(48.6)		101(52.3)		72(48.7)	
O level secondary	20(3.9)	3(2.9)		2(1)		3(2)	
A level secondary	81(15.9)	25(24.3)		17(8.8)		24(16.2)	
College/University	33(6.5)	7(6.8)		15(7.8)		11(7.4)	
**Physical activity**							
Inactive	225(44.1)	60(58.3)	**0.005**	95(49.2)	**0.157**	80(54.1)	**0.015**
Moderately active	86(16.9)	12(11.7)		32(16.6)		21(14.2)	
Highly active	199(39)	31(30.1)		66(34.2)		47(31.8)	
**Dietary diversity (DDS)**							
*Mean (SD)*	*4*.*3(2*.*2)*						
Adequate (≥4 DDS)	277(54.3)	62(60.2)	**0.180**	83(43.1)	**<0.001**	71(48)	**0.066**
Inadequate(<4DDS)	233(45.7)	41(39.8)		110(56.9)		77(52)	

^a^ Chi-square test comparing obesity prevalence

^b^ Based on criteria of BMI ≥30kg/m^2^

^c^ Based on WHO criteria of WHR≥0.90 in males and WHR≥0.85 in females

^d^ Based on criteria of WC≥102 cm in males and WC≥88 cm in females

### Association between dietary diversity with overweight/obesity

In **Table 1**, consumption of adequate dietary diversity (DDS ≥4) was achieved by 54.3% of the participants. Nearly all (93.7%) participants had consumed foods made of cereals, and 33.9% consumed food items made from legumes. This was followed by consumption of milk and dairy products (43.9%), meat (30.2%), fatty foods (29.0%), and very few consumed eggs (4.3%). Consumption of vegetables (60.6%) was higher than fruits (16.9%). Males (54.4%) consumed more milk than females (34.6), while females (68.8%) consumed more vegetables than males (51.5%), as shown in **Table 2**. Moreover, in a bivariate analysis, consumption of adequate dietary diversity was associated with abdominal obesity by WHR (*p*<0.001), but not abdominal obesity by WC (*p* = 0.066) and general obesity by BMI (*p* = 0.180) as shown in **Table 1**. Furthermore, we examined the association between DDS terciles with overweight and obesity measures by controlling for potential confounders. Results show that higher DDS tercile was inversely associated with abdominal obesity measured by WHR among male and female participants. In **Table 3**, males who were on the middle DDS tercile have high prevalence of overweight (PR = 2.03; 95% CI, 1.23–3.33) than those in the lower DDS tercile. However, the association becomes not significant when controlled for all potential confounders in the model (APR = 1.51; 95% CI, 0.91–2.49). In addition, males who were on the higher DDS tercile have low prevalence of abdominal obesity by WHR (APR = 0.42; 95% CI, 0.22–0.77). On the other hand in **Table 4**, female participants who were in higher DDS tercile have low prevalence of abdominal obesity by WHR (APR = 0.63; 95% CI, 0.41–0.94). In contrast, female subjects who were in higher DDS tercile have increased prevalence of general obesity (APR = 1.62; 95% CI, 1.13–2.33). In general, this study shows that there was no significant association between DDS and abdominal obesity using WC for both male and female (**Tables 3 and 4**).

**Table 2:** Dietary diversity and consumption of food groups between genders of the participants in Monduli District, Tanzania.

Variables	Total(*n* = 510)	Male (*n* = 241)	Female (*n* = 269)	*P-*value^a^
	n(%)			
**Food groups**				
Cereals	478(93.7)	221(91.7)	257(95.5)	0.074
Roots and tubers	118(23.1)	54(22.4)	64(23.8)	0.711
Legumes and nuts	173(33.9)	97(40.2)	76(28.3)	0.004
Fish	86(16.9)	33(13.7)	53(19.7)	0.070
Milk	224(43.9)	131(54.4)	93(34.6)	<0.001
Eggs	22(4.3)	9(3.7)	13(4.8)	0.542
Fats and oils	148(29.0)	77(32.0)	71(26.4)	0.168
Sweets	114(22.4)	62(25.7)	52(19.3)	0.083
Condiments and beverages	311(61.0)	156(64.7)	155(57.6)	0.100
Vegetables	309(60.6)	124(51.5)	185(68.8)	<0.001
Fruits	86(16.9)	42(17.4)	44(16.4)	0.747
Meat	154(30.2)	84(34.9)	70(26.0)	0.030
**DDS index**				
Lower tercile	233(45.7)	103(42.7)	130(48.3)	0.145
Middle tercile	145(28.4)	66(27.4)	79(29.4)	
Higher tercile	132(25.8)	72(29.8)	60(22.3)	

a-Chi-square test

**Table 3:** Multivariate Poisson regressions (PR-Prevalence ratio and 95% confidence intervals) for dietary diversity (DDS Terciles) with overweight and obesity among male participants in Monduli district, Tanzania (*N* = 241).

Variables		Lower Tercile	Middle Tercile	Higher Tercile
		PR (95% CI)	PR (95% CI)	PR (95% CI)
**Overweight (BMI)**^**a**^				
	Model 0	1.0	2.03 (1.23–3.33)**	1.22(0.68–2.15)
	Model 1	1.0	1.93(1.19–3.12)**	1.29(0.75–2.21)
	Model 2	1.0	1.68(1.02–2.78)*	1.28(0.71–2.29)
	Model 3	1.0	1.51(0.91–2.49)	1.31(0.75–2.27)
**General obesity (BMI)**^**b**^				
	Model 0	1.0	4.68(0.49–44.06)	2.86(0.26–30.96)
	Model 1	1.0	4.63(0.56–38.01)	3.48(0.27–44.0)
	Model 2	1.0	5.34(0.74–38.34)	1.57(0.03–67.32)
	Model 3	1.0	4.32(0.61–30.77)	2.48(0.06–102.1)
**Abdominal obesity (WHR)**^**c**^				
	Model 0	1.0	0.63(0.37–1.06)	0.46(0.26–0.83)**
	Model 1	1.0	0.63(0.42–0.97)*	0.62(0.38–1.01)
	Model 2	1.0	0.65(0.32–0.96)*	0.44(0.24–0.79)**
	Model 3	1.0	0.57(0.35–0.93)*	0.42(0.22–0.77)**
**Abdominal obesity (WC)**^**d**^				
	Model 0	1.0	0.78(0.15–4.14)	1.07(0.25–4.65)
	Model 1	1.0	0.83(0.17–4.01)	1.61(0.42–6.24)
	Model 2	1.0	0.65(0.09–4.82)	0.62(0.17–2.18)
	Model 3	1.0	0.61(0.13–2.73)	0.23(0.02–2.53)

*P < .05

**P < .01

***P<0.001; CI = confidence interval; PR = Prevalence ratio, 1.0 = Reference category

Model 0 crude

Model 1 adjusted for age

Model 2 adjusted for income, education, physical activity

Model 3 adjusted for all confounding factors

^a^ Based on criteria of BMI ≥25–30 kg/m^2^

^b^ Based on criteria of BMI ≥30 kg/m^2^

^c^ Based on criteria of WHR≥0.90 in males

^d^ Based on criteria of WC≥102 cm in males

**Table 4:** Multivariate Poisson regressions (PR-Prevalence ratio and 95% confidence intervals) for dietary diversity (DDS Terciles) with overweight and obesity among female participants in Monduli district, Tanzania (*N* = 269).

Variables		Lower Tercile	Middle Tercile	Higher Tercile
		PR (95% CI)	PR (95% CI)	PR (95% CI)
**Overweight (BMI)**^**a**^				
	Model 0	1.0	1.34(0.82–2.18)	1.12(0.64–1.98)
	Model 1	1.0	1.39(0.85–2.26)	1.20(0.67–2.15)
	Model 2	1.0	1.34(0.82–2.17)	1.03(0.58–1.82)
	Model 3	1.0	1.37(0.85–2.22)	1.01(0.55–1.86)
**General obesity (BMI)**^**b**^				
	Model 0	1.0	1.15(0.77–1.71)	1.57(1.08–2.27)*
	Model 1	1.0	1.10(0.76–1.61)	1.73(1.22–2.45)**
	Model 2	1.0	1.16(0.78–1.74)	1.49(0.99–2.24)
	Model 3	1.0	1.10(0.77–1.58)	1.62(1.13–2.33)**
**Abdominal obesity (WHR)**^**c**^				
	Model 0	1.0	0.87(0.67–1.15)	0.50(0.32–0.77)**
	Model 1	1.0	0.91(0.69–1.19)	0.59(0.38–0.92)*
	Model 2	1.0	0.98(0.73–1.29)	0.60(0.39–0.93)*
	Model 3	1.0	0.97(0.74–1.27)	0.63(0.41–0.94)*
**Abdominal obesity (WC)**^**d**^				
	Model 0	1.0	0.81(0.61–1.08)	0.89(0.66–1.19)
	Model 1	1.0	0.82(0.63–1.06)	1.08(0.83–1.42)
	Model 2	1.0	0.86(0.65–1.15)	0.97(0.71–1.33)
	Model 3	1.0	0.84(0.65–1.08)	1.06(0.80–1.39)

*P < .05

**P < .01

***P<0.001; CI = confidence interval; PR = Prevalence ratio, 1.0 = Reference category

Model 0 crude

Model 1 adjusted for age only

Model 2 adjusted for income, education, physical activity

Model 3 adjusted for all confounding factors

^a^ Based on criteria of BMI ≥25–30 kg/m^2^

^b^ Based on criteria of BMI ≥30 kg/m^2^

^c^ Based on criteria of WHR≥0.85 in females

^d^ Based on criteria of WC≥88 cm in females

## Discussion

This study was conducted to examine the importance of consumption of a diversified diet on overweight and obesity among adults living in pastoral communities in Monduli district in Tanzania. The prevalence of obesity depends on the criteria applied; we considered both general and abdominal obesity measures because these may have different implications in the emergence of NCDs. We found that the prevalence of abdominal obesity when measured by WHR (37.8%) or WC (29.1%) was higher than that of general obesity measured by BMI (20.2%). The present study noted that consuming a diversified diet was linked to the reduction of abdominal obesity measured by WHR. This finding is similar to previous studies that assessed the relationship between dietary diversity and health outcomes such as obesity [20]. However, in our study, based on the stratified analysis between male and female, and when considering different types of confounders, inconsistent positive associations were found between dietary diversity and overweight and general obesity by BMI. This finding support the previous evidence that abdominal obesity measures are more predictive in diet-related NCDs, and are good measure of abdominal and intra-abdominal fat deposition than BMI measure [34]. Overall, this finding highlights the importance of consumption of a diversified diet in the prevention against abdominal obesity and other associated NCDs such as cardiovascular diseases and type 2 diabetes in pastoral communities in Tanzania.

This study revealed that the positive association between DDS and general obesity (BMI) was only evident among female participants signifying the stronger effect of dietary diversity on weight status of females. These may be due to the fact that females are more obese than males in all obesity measures, as shown in **Table 1**. The underlying mechanism that explain the positive link between DDS and increase in weight is unclear, but increased consumption of energy dense foods like starchy foods with less fruits have been reported [35]. In addition, it is well known that consumption of diversified and varieties of food items is a predictor of dietary adequacy and energy intake which increase body weight and finally lead to obesity [17]. Even though we controlled for other confounders in this study, it may have been important to adjust the association with dietary energy intake which may attenuate, and thus possibly contributing toward this association. In comparison, Azadbakht et al, [36] reported that adults who consumed a more diversified diet had a higher BMI than adults who consumed a less diverse diet. Therefore, in any case promotion of dietary diversity should be done in parallel with caution for this community, particularly among females who have shown high prevalence of obesity.

This study also shows that there was a positive association between DDS and overweight among male participants only. However; this association was not evident after controlling for other confounders implying that this association could be a consequence of other factors and not exclusively the result of higher dietary diversity. These factors could be physical activity, income, age or education. Physical activity factor has potential role among males in this study. Unlike females, males usually walk long distances daily to herd cattle, while females usually stay home and perform light work, most of which are of moderate energy cost [7]. In other words, dietary diversity combined with physical activity may be capable of ensuring healthy weight among males. In this study, we found that the prevalence of obesity was significantly higher among inactive than active participants for both genders. However, in this study, we adjusted our analysis for physical activity factor. In comparison, Karimbeiki et al, [37] have found a positive association between DDS and increase in weight among Iranian adults; however, the association became slightly weaker after adjusting for potential confounding factors like physical activity.

Overall, we found that more than half of the participants have consumed an adequate diversified diet of at least 4 food groups out of 12. However, higher dietary diversity does not necessarily mean healthy eating pattern [28]. Our findings show that the diet consumed in pastoral communities was dominated by cereals or legumes staples, with moderate intake of animal-sourced foods, and very little fruits. This implies a poor diet quality, which leads to higher calories consumption, low in protein, minerals and vitamins. The health impact of this type of diet is usually impaired growth and cognitive development, and reduced body’s resistance to infections. Such a type of diet was also reported among farming households in rural Tanzania [32]. Perhaps prolonged drought at the time of this study may be the reason for this type of diet [38]. It is important to note that dietary diversity may vary with season, and rural population depends their diet from rain-fed agriculture. This means that at certain times of the year food is available in abundance, while at other times there is little foods. This therefore warranting for additional longitudinal investigations to identify the habitual dietary pattern that may be beneficial or detrimental to healthy weight in this population.

Some important limitations should be considered in this study. Due to the cross-sectional nature of this study, one cannot establish the causation between DDS and overweight/obesity. We derived the DDS from a one-day recall period, which can be affected be season. However, a single 24-hour recall is regarded as the good reference period to derive the DDS and longer reference periods may lead to imperfect recall [14, 39]. The information about the portion sizes consumed was incomplete for most of the participants, therefore, we were unable to determine the dietary energy consumed. Furthermore, this study may be limited by other residual confounding which are unmeasured during data collection. However, we managed to adjust the association for some important confounders to test the association of DDS with obesity. Apart from being one of the few studies ever conducted in pastoral communities in Tanzania, this study has some important strengths. Key outcomes such as weight, height, waist and hip circumferences were objectively measured rather than self-reported.

## Conclusion

More than half of adults in pastoral communities consumed an adequate diversified diet. This diet was dominated by cereals, vegetables or legumes staples, with moderate intake of animal-sourced foods, and very little fruits. This study suggests that consuming a diversified diet is associated with a reduction of the abdominal obesity in this population. Given the inconsistent findings on association between dietary diversity with overweight and general obesity, dietary diversity should be promoted with careful consideration. Moreover, communities should be well informed on making nutritious food choices rather than diversity alone. In particular, there should be a promotion of consumption of fruits, vegetables and animal-sourced foods such as eggs, meat, fish and poultry. Additional formative research are required to determine the quantity of foods consumed to further understand the associations with obesity and associated NCDs.

## Supporting information

S1 Data (XLS)

S1 Questionnaires (DOCX)

## Abbreviations

APR: Adjusted prevalence ratio
BMI: Body mass index
DDS: Dietary diversity score
FG: Food groups
NCDs: Non-communicable diseases
PA: Physical activity
PR: Prevalence ratio
WHR: Waist to hip ratio
WC: Waist circumference
WHO: World health organization
