# Supplementary material for: Association between dietary diversity with overweight and obesity: A cross-sectional study conducted among pastoralists in Monduli District in Tanzania
Source: PLoS One. 2021 Jan 13;16(1):e0244813. doi: 10.1371/journal.pone.0244813 (PMC7806168; doi:10.1371/journal.pone.0244813)
Supplement: S1 Questionnaires — (DOCX) [file pone.0244813.s002.docx]

**Questionnaires**

**PART 1: SOCIO-DEMOGRAPHIC INFORMATION**

| **Questionnaire No:** | | | | | | | | | | | | |
| --- | --- | --- | --- | --- | --- | --- | --- | --- | --- | --- | --- | --- |
| **Participant Identification Number** └─┴─┴─┘└─┴─┴─┘└─┴─┴─┘ | | | | | | | | | | | | |
| **1-Regional Characteristics** | | | | | | | | | | | | |
| 1.1 | Enumerator’s name | |  | | | 1.2 | | Interview date | |  | | |
| 1.3 | District name | |  | | | 1.4 | | Is Urban/Rural  1= Rural, 0= Urban | |  | | |
| 1.5 | Name of village/town/colony | |  | | | 1.6 | | Is this village/colony located in desert/arid area? | | 1= Yes, desert/arid area  0= No | | [_______] |
| **2-Household Characteristics** | | | | | | | | | | | | |
| 2.1 | Name of respondent |  | | | | 2.2 | Gender of respondent | | 1=Male, 0=Female | | [_______] | |
| 2.3 | What is relationship of the respondent with the head of household?  1=Self, 2=Spouse (Wife/ Husband), 3=Other member of household | | | | [___] | 2.4 | Phone number of the respondent (optional) | | | |  | |
| 2.5 | What is gender of the head of household? | | | 1=Male, 0=Female | [___] | 2.6 | What is the marital status of the head of household?  1=Unmarried, 2=Married, 3=Divorced/Separated, 4=Widow/Widower | | | | [______] | |
| 2.7 | What is the highest level of education?  1= Never been to school, 2= Primary (1-5), 3=Primary completed, 4=Secondary (6-10), 5= Secondary completed, 6=Higher secondary (11-12), 7=Higher secondary completed, 8=Graduation/Post graduation, 9= Other__________________ | | | | | | | | | | [______] | |
| 2.8 | What is the highest level of education of the spouse?  1= Never been to school, 2= Primary (1-5), 3=Primary completed, 4=Secondary (6-10), 5= Secondary completed, 6=Higher secondary (11-12), 7=Higher secondary completed, 8=Graduation/Post graduation, 9= Other__________________ | | | | | | | | | | [______] | |
| 2.9 | What Is the religion of this household? (Code: 1= Buddhism, 2=Christian, 3=Hindu, 4=Islam, 5=Others (-------) | | | | | | | | | | | |
| Male |  | | | | | | | | | | | |

| **3-Livestock Ownerships** | | | | | | | | | | | | | | | | | | | | | | | | | | | | | | | | | | |
| --- | --- | --- | --- | --- | --- | --- | --- | --- | --- | --- | --- | --- | --- | --- | --- | --- | --- | --- | --- | --- | --- | --- | --- | --- | --- | --- | --- | --- | --- | --- | --- | --- | --- | --- |
| 3.1 | Do you own livestock/poultry? | | | | | | | | | | | | 1= Yes, 0 = No | | | | | │______│ | | | | | | | | | | | | | | | | |
|  | | **4.1a How many heads do you own today?** | | | | | **4.1b How many heads did you own in the past season?** | | | | | | | | | | | **4.1c How many heads did you own same season last year?** | | | | | | | | | | | | | | | | |
| Cows/ Buffalos | |  | | │______│ | | |  | | | │______│ | | | | | | | |  | | | | | │______│ | | | | | | | | | | | |
| Goats/ Sheep | |  | | │______│ | | |  | | | │______│ | | | | | | | |  | | | | | │______│ | | | | | | | | | | | |
| Poultry | |  | | │______│ | | |  | | | │______│ | | | | | | | |  | | | | | │______│ | | | | | | | | | | | |
| What is status of availability of following items for livestock?  1= Sufficiently available, 2= Less than sufficient available, 3= Least/not available | | | | | | | | | | | | | | 3.3.1 | | | Water | | | | | │__│ | | | | 3.3.2 | | | | Shelter | | | | │__│ |
|  |  |  |  |  |  |  |  |  |  |  |  |  |  | 3.3.3 | | | Fodder | | | | | │__│ | | | | 3.3.4 | | | | Vaccinations/medicines | | | | │__│ |
| **4-Assets Ownership** | | | | | | | | | | | | | | | | | | | | | | | | | | | | | | | | | | |
| Do/did own following assets? ***Ask for each asset below***. *(1= Yes, 0= No)* | | | | | | | | | | | | | | | | | | | | | | | | | | | | | | | | | | |
| **Assets^^[[1]](#footnote-1)^^** | | | **6 months ago** | | | **Currently** | | | | | | | | | **Assets** | | | | | **6 months ago** | | | | | | | | | **Currently** | | | | | |
| Electricity | | | 4.1 | │_____│ | | 4.5 | | | │_____│ | | | | | | Radio/TV | | | | | 4.9 | | | | | │_____│ | | | | 4.13 | | | | │______│ | |
| Sewing machine | | | 4.2 | │_____│ | | 4.6 | | | │_____│ | | | | | | Telephone/  Mobile | | | | | 4.10 | | | | | │_____│ | | | | 4.14 | | | | │______│ | |
| Tractor | | | 4.3 | │_____│ | | 4.7 | | | │_____│ | | | | | | Refrigerator | | | | | 4.11 | | | | | │_____│ | | | | 4.15 | | | | │______│ | |
| Plough/  Agri. tools | | | 4.4 | │_____│ | | 4.8 | | | │_____│ | | | | | | Motorbike/  bicycle | | | | | 4.12 | | | | | │_____│ | | | | 4.16 | | | | │______│ | |
| **5- Sources of Livelihoods/Income** | | | | | | | | | | | | | | | | | | | | | | | | | | | | | | | | | | |
|  | | | | | | | | **Currently** | | | | | | | | | | | **6 months ago** | | | | | | | | | | | | | | | |
| What are/were **two main sources of livelihood** for your household**?**  *(use codes below)* | | | | | | | | 5. 1 | | | **1st** │_____│ | | | | | | | | 5. 3 | | | | | **1st** │_____│ | | | | | | | | | | |
|  |  |  |  |  |  |  |  | 5. 2 | | | **2nd** │_____│ | | | | | | | | 5. 4 | | | | | **2nd** │_____│ | | | | | | | | | | |
| **Livelihood sources:** 1 = Sale of food/cash crops, 2= Sale of vegetables/fruits, 3 = Agricultural wage labour, 4 = Non-agricultural wage labour, 5 = Small business (self-employed), 6=Medium to large business (self-employed), 7 = Government employee, 8 = NGO/private employee, 9= Professional (doctor, engineer, lawyers), 10= Handicrafts, 11 = Sale of livestock, 12= Sale of animal products, 13 = Petty trade, 14 = Pension/ allowances, 15 = Remittances (domestic/foreign), 16 = charity/zakat/gifts, 17 = Sale of firewood/grass/charcoal sales, 18=Fishery/forestry worker, 19=Other (specify)_________________, 99 = No 2^nd^ or 3^rd^ source of income | | | | | | | | | | | | | | | | | | | | | | | | | | | | | | | | | | |
|  | | | | | | | | | | | | | | | | **Currently** | | | | | | | | | | | | **6 months ago** | | | | | | |
| How much **share** of total monthly income is/was **from off-farm (non-agriculture)** sources? **(Write in %)** | | | | | | | | | | | | | | | | 5.5 | | | | | │______│ | | | | | | | 5.6 | | | | │______│ | | |
| **6- Expenditure** | | | | | | | | | | | | | | | | | | | | | | | | | | | | | | | | | | |
| How much did your spend **LAST MONTH** on food and other items? | | | | | | | | | | | | | | | | | | | | | | | | | | | | | | | | | | |
| **Item** | | | | | | **Amount** (in local currency) | | | | | | **Item** | | | | | | | | | | | | | | | | | | | **Amount** | | | |
| Food | | | | | 6.1 | │______│ | | | | | | Non-food items (health, education, clothing, shoes, transport, utility bills, ceremonies, livestock fodder/feed, reimbursement of debt etc) | | | | | | | | | | | | | | | 6.2 | | | | │______│ | | | |

| **7-Migration** | | | | | | | |
| --- | --- | --- | --- | --- | --- | --- | --- |
| 7.1 | Did you or members of household migrate to any other area during **past 6 months**? | | | 1=Yes, seasonal migration  2=No>> **Next Section** | | [_____] | |
| What were the three main reasons for this migration? **See codes below** | | 7.2.1 | [_____] | 7.2.2 | [_____] | 7.2.3 | [_____] |

| 1=Less livelihood opportunities in the area, 2=Loss of livelihood, 3=lack of drinking water 4=lack of fodder/grazing land for livestock 5=Diseases/illness of household member 6=Non availability of the irrigation water, 7= Other (specify)___________________ |
| --- |
| 8. In the past 30 days, did you suffered/ diagnosed with the following diseases? Select the right choice  1. Malaria  2. Diarrhea  3. Woms  4. others (Specify)……… |

**PART 2: PHYSICAL ACTIVITY**

| **Questions** | **Descriptions** | **Response** |
| --- | --- | --- |
| **Working** | |  |
| P1 | Does your work involve vigorous-intensity activity that causes large increases in breathing or heart rate like [carrying or lifting heavy loads, digging or construction work] for at least 10 minutes continuously? ***If No move to P4*** | 1= Yes 0= No. \|____\| |
| P2 | In a typical week, on how many days do you do vigorous- intensity activities as part of your work? | [_____] days |
| P3 | How much time do you spend doing vigorous-intensity activities at work on a typical day? | Hrs min  [__[___]:[__[___] |
| p4 | Does your work involve moderate-intensity activity, that causes small increases in breathing or heart rate such as brisk walking [walking with animals] for at least 10 minutes continuously?  ***If No move to P7*** | 1= Yes 0= No. \|____\| |
| p5 | In a typical week, on how many days do you do moderate-intensity activities as part of your work? | [_____] days |
| p6 | How much time do you spend doing moderate-intensity activities at work on a typical day? | Hrs min  [__[___]:[__[___] |
| **Transport to and from places** | |  |
| P7 | Do you walk or use for at least 10 minutes continuously to get to and from places? ***If No move to P10*** | 1= Yes 0= No. \|____\| |
| P8 | In a typical week, on how many days do you walk or bicycle for  at least 10 minutes continuously to get to and from places? | [_____] days |
| P9 | How much time do you spend walking or bicycling for travel on a  typical day? | Hrs min  [__[___]:[__[___] |
| **Recreational Activities** | |  |
| P10 | Do you do any vigorous-intensity sports, fitness or recreational  (leisure) activities that cause large increases in breathing or heart rate like [running or football] for at least 10 minutes continuously? ***If No move to P13*** | 1= Yes 0= No. \|____\| |
| P11 | In a typical week, on how many days do you do vigorous-intensity sports, fitness or recreational (leisure) activities? | [_____] days |
| P12 | How much time do you spend doing vigorous-intensity sports,  fitness or recreational activities on a typical day? | Hrs min  [__[___]:[__[___] |
| P13 | Do you do any moderate-intensity sports, fitness or recreational  (leisure) activities that cause a small increase in breathing or heart rate such as brisk walking, [cycling, swimming, volleyball]  for at least 10 minutes continuously? | 1= Yes 0= No. \|____\| |
| P14 | In a typical week, on how many days do you do moderate-  intensity sports, fitness or recreational (leisure) activities? | [_____] days |
| P15 | How much time do you spend doing moderate-intensity sports, fitness or recreational (leisure) activities on a typical day? | Hrs min  [__[___]:[__[___] |

**PART 3: DISEASE HISTORY**

| **History of Raised Blood Pressure (Hypertension)** | | | | | | |  |
| --- | --- | --- | --- | --- | --- | --- | --- |
| **Question** | | **Response** | | | **Code** | |  |
| Have you ever had your blood pressure measured by a doctor or other health worker? | | Yes | 1 | | H1 | |  |
|  |  | No | 2 *If No, go to H6* | |  |  |  |
| Have you ever been told by a doctor or other health worker that you have raised blood pressure or hypertension? | | Yes | 1 | | H2a | |  |
|  |  | No | 2  *If No, go to H6* | |  |  |  |
| Were you first told in the past 12 months? | | Yes | 1 | | H2b | |  |
|  |  | No | 2 | |  |  |  |
| In the past two weeks, have you taken any drugs (medication) for raised blood pressure prescribed by a doctor or other health worker? | | Yes | 1 | | H3 | |  |
|  |  | No | 2 | |  |  |  |
| Have you ever seen a traditional healer for raised blood pressure or hypertension? | | Yes | 1 | | H4 | |  |
|  |  | No | 2 | |  |  |  |
| Are you currently taking any herbal or traditional remedy for your raised blood pressure? | | Yes | 1 | | H5 | |  |
|  |  | No | 2 | |  |  |  |
| **History of Diabetes** | | | | | | | |
| Have you ever had your blood sugar measured by a doctor or other health worker? | Yes | | | 1 | | H6 | |
|  | No | | | 2 *If No, go to H12* | |  |  |
| Have you ever been told by a doctor or other health worker that you have raised blood sugar or diabetes? | Yes | | | 1 | | H7a | |
|  | No | | | 2  *If No, go to H12* | |  |  |
| Were you first told in the past 12 months? | Yes | | | 1 | | H7b | |
|  | No | | | 2 | |  |  |
| In the past two weeks, have you taken any drugs (medication) for diabetes prescribed by a doctor or other health worker? | Yes | | | 1 | | H8 | |
|  | No | | | 2 | |  |  |
| Are you currently taking insulin for diabetes prescribed by a doctor or other health worker? | Yes | | | 1 | | H9 | |
|  | No | | | 2 | |  |  |
| Have you ever seen a traditional healer for diabetes or raised blood sugar? | Yes | | | 1 | | H10 | |
|  | No | | | 2 | |  |  |
| Are you currently taking any herbal or traditional remedy for your diabetes? | Yes | | | 1 | | H11 | |
|  | No | | | 2 | |  |  |
|  |  | | |  | |  | |

**PART 4: ANTHROPOMETRIC AND PHYSICAL MEASUREMENT**

| **Blood Pressure (BP)** | | | | | | |  |  |
| --- | --- | --- | --- | --- | --- | --- | --- | --- |
| **Question** | | **Response** | | | | **Code** |  |  |
| Interviewer ID | |  | | └─┴─┴─┘ | | M1 |  |  |
| Device ID for blood pressure | |  | | └─┴─┘ | | M2 |  |  |
| Cuff size used | | Small | | 1 | | M3 |  |  |
|  |  | Medium | | 2 | |  |  |  |
|  |  | Large | | 3 | |  |  |  |
| Reading 1 | | Systolic ( mmHg) | | └─┴─┴─┘ | | M4a |  |  |
|  |  | Diastolic (mmHg) | | └─┴─┴─┘ | | M4b |  |  |
| Reading 2 | | Systolic ( mmHg) | | └─┴─┴─┘ | | M5a |  |  |
|  |  | Diastolic (mmHg) | | └─┴─┴─┘ | | M5b |  |  |
| Reading 3 | | Systolic ( mmHg) | | └─┴─┴─┘ | | M6a |  |  |
|  |  | Diastolic (mmHg) | | └─┴─┴─┘ | | M6b |  |  |
| During the past two weeks, have you been treated for raised blood pressure with drugs (medication) prescribed by a doctor or other health worker? | | Yes | | 1 | | M7 |  |  |
|  |  | No | | 2 | |  |  |  |
| **Height and Weight** | | | | | | |  |  |
| **For women:** Are you pregnant? | | Yes | | 1 *If Yes, go to M 16* | | M8 |  |  |
|  |  | No | | 2 | |  |  |  |
| Interviewer ID | |  | | └─┴─┴─┘ | | M9 |  |  |
| Device IDs for height and weight | | Height | | └─┴─┘ | | M10a |  |  |
|  |  | Weight | | └─┴─┘ | | M10b |  |  |
| Height | | in Centimetres (cm) | | └─┴─┴─┘. └─┘ | | M11 |  |  |
| Weight  *If too large for scale 666.6* | | in Kilograms (kg) | | └─┴─┴─┘.└─┘ | | M12 |  |  |
| **Waist** | | | | | | |  |  |
| Device ID for waist | |  | | └─┴─┘ | | M13 |  |  |
| Waist circumference | | in Centimetres (cm) | | └─┴─┴─┘.└─┘ | | M14 |  |  |
| **Hip Circumference and Heart Rate** | | | | | | | | |
| Hip circumference | | in Centimeters (cm) | | └─┴─┴─┘.└─┘ | | M15 | | |
| Heart Rate | |  | |  | |  | | |
| Reading 1 | | Beats per minute | | └─┴─┴─┘ | | M16a | | |
| Reading 2 | | Beats per minute | | └─┴─┴─┘ | | M16b | | |
| Reading 3 | | Beats per minute | | └─┴─┴─┘ | | M16c | | |

**PART 5: BIOCHEMICAL MEASUREMENT**

| **Blood Glucose** | | | |
| --- | --- | --- | --- |
| **Question** | **Response** | | **Code** |
| During the past 12 hours have you had anything to eat or drink, other than water? | Yes | 1 | B1 |
|  | No | 2 |  |
| Technician ID |  | └─┴─┴─┘ | B2 |
| Device ID |  | └─┴─┘ | B3 |
| Time of day blood specimen taken (24 hour clock) | Hours : minutes | └─┴─┘: └─┴─┘  hrs mins | B4 |
| Fasting blood glucose  *[CHOOSE ACCORDINGLY: MMOL/L OR MG/DL]* | mmol/l | └─┴─┘. └─┴─┘ | B5 |
|  | mg/dl | └─┴─┴─┘.└─┘ |  |
| Today, have you taken insulin or other drugs (medication) that have been prescribed by a doctor or other health worker for raised blood glucose? | Yes | 1 | B6 |
|  | No | 2 |  |

**PART 6: 24-hours Dietary Recall**

|  |  |
| --- | --- |
|  | **Instructions** |
|  | Now I’d like to ask you about foods and drinks that you ate or drank yesterday during the day or night, whether you ate at home or anywhere else.  I am interested in all the foods you ate, including those that were combined with other foods. For example, if you had a soup made with carrots, potatoes and meat, you should mention all the individual foods in that soup (that is carrots, potatoes and meat). However, if you consumed only the broth of a soup, but not the meat or vegetable, do not mention the meat or vegetable.  As I ask you about foods and drinks, please think of foods and drinks you had as snacks or small meals, food and meals that you ate when you got up in the middle of the night, as well any main meals as during day. Please also remember foods you may have eaten while preparing meals or preparing food for others, as well as foods you ate way from home. |

**Participant Identification Number** └─┴─┴─┘└─┴─┴─┘└─┴─┴─┘

| **Household member number** | | | | | | | | | | | | | |
| --- | --- | --- | --- | --- | --- | --- | --- | --- | --- | --- | --- | --- | --- |
| 01 | | 02 | 03 | | 04 | 05 | 06 | | 07 | 08 | 09 | | 10 |
|  | | | | | | | | | | | | | |
|  | **9a** | | | **9b** | | **9c** | | **9d***** | | | | **9e** | |
|  | **The questions on consumption** | | | **If yes, what did you eat?** | | **Anything else?** | | **Ask about the food/dish ingredients and details** | | | | | |
|  |  | | | *Lists below all foods below, for mixed dishes mention all ingredients for that dish* | |  | | What was the (food) you (ate/drank) made of? What food ingredients were in the (meal or dish)? | | | | Did it have any other ingredients? [If yes] What were they? | |
| 1 | Did you have anything to eat or drink when you woke up?  1=Yes Ask 9b to 9e  2=No Go to 2 | | | 1.  2.  3.  4.  5.  6.  7.  8.  9.  10. | | 1.  2.  3.  4.  5. | | *1*  2.  3.  4.  5.  6.  7.  8  9.  10. | | | |  | |
|  | Now, I’m going to ask you more details about the foods and beverages you just listed. I want you tell me “when”, “how much” of these foods you ate yesterday.  When I ask about amounts, you can use these measuring guides and food pictures for the  size or amounts of foods. *Use approximate measures of Teaspoon(s), Tablespoon (s) or cup (s), 1/3, ¼ , ½ pieces or 1, 2, 3, …piece(s), from the food models.* | | | | | | | | | | | | |
|  | **9f. How much of these foods did you eat?** | | | | |  | | **9g. When were these foods consumed** | | | | | |
|  | Food list***  1.  2.  3.  4.  5.  6.  7.  8.  9.  10. | | | Amounts | |  | | **Time** | | | |  | |
|  |  | | |  | |  | |  | | | |  | |
| 2 | Did you have anything to eat or drink later in the morning?  1=Yes Ask 9b to 9e  2=No Go to 3 | | | 1.  2.  3.  4.  5.  6.  7.  8.  9.  10. | | 1.  2.  3.  4.  5. | |  | | | |  | |
|  | **9f. How much of these foods did you eat?** | | | | |  | | **9g. What time did you eat or drink the food (s)** | | | | | |
|  | Food list*** | | | Amounts | |  | | Time: | | | |  | |
|  |  | | |  | |  | |  | | | |  | |
| 3 | Did you eat or drink anything at mid-day?  1=Yes Ask 9b to 9e  2=No Go to 4 | | | 1.  2.  3.  4.  5.  6.  7.  8.  9.  10. | | 1.  2.  3.  4.  5. | |  | | | |  | |
|  | **9f. How much of these foods did you eat?** | | | | |  | | **9g. What time did you eat or drink the food (s)** | | | | | |
|  | Food list*** | | | Amounts | |  | | Time: | | | |  | |
|  |  | | |  | |  | |  | | | |  | |
| 4 | Did you have anything to eat or drink during the afternoon  1=Yes Ask 9b to 9e  2=No Go to 5 | | | 1.  2.  3.  4.  5.  6.  7.  8.  9.  10. | | 1.  2.  3.  4.  5. | |  | | | |  | |
|  | **9f. How much of these foods did you eat?** | | | | |  | | **9g. What time did you eat or drink the food (s)** | | | | | |
|  | Food list*** | | | Amounts | |  | | Time: | | | |  | |
|  |  | | |  | |  | |  | | | |  | |
| 5 | Did you have anything to eat in the evening?  1=Yes Ask 9b to 9e  2=No Go to 6 | | | 1.  2.  3.  4.  5.  6.  7.  8.  9.  10. | | 1.  2.  3.  4.  5. | |  | | | |  | |
|  | **9f. How much of these foods did you eat?** | | | | |  | | **9g. What time did you eat or drink the food (s)** | | | | | |
|  | Food list*** | | | Amounts | |  | | Time: | | | |  | |
|  |  | | |  | |  | |  | | | |  | |
| 6 | Did you have anything else to eat or drink in the evening before going to bed or during the night?  1=Yes Ask 9b to 9e  2=No | | | 1.  2.  3.  4.  5.  6.  7.  8.  9.  10. | | 1.  2.  3.  4.  5. | |  | | | |  | |
|  | **9f. How much of these foods did you eat?** | | | | |  | | **9g. What time did you eat or drink the food (s)** | | | | | |
|  | Food list*** | | | Amounts | |  | | Time: | | | |  | |
|  |  | | |  | |  | |  | | | |  | |
|  |  | | |  | |  | |  | | | |  | |
| 9h  (25) | Was the day yesterday a typical day for you in terms of the foods, amount and frequency of eating about usual, less than usual, or more than usual?  (1) Usual (Go to 9.)  (2) Less than usual (Go to 9h.2)  (3) More than usual (Go to 9h.3) | | | 9h.2 (26) What is the main reason the amount you ate yesterday was less than usual?  (1) Sickness  (2) Short of money  (3) Traveling (4) At a social function, special meal or on a special day (5) On vacation (6) Too busy (7) Not hungry (8) Dieting  (9) Fasting (10) Bored (  11) Stressed (12) Other reason:____ | | 8b. (27) What is the main reason the amount you ate yesterday was more than usual? (1) Traveling (2) At a social function, special meal, or on a special day (3) On vacation or day off  (4) Very hungry  (5) Bored or stressed  (6) Some other reason: ------ | | 9.i (28) How could you describe your current dietary habit? [Show card] (1) No special diet, I eat almost everything  (2) Vegetarian  (3) Special diet: ………….. | | | |  | |

1. [↑](#footnote-ref-1)
